# Characterization and clinical relevance of PDGFRA pathway copy number variation gains across human cancers

**DOI:** 10.1007/s00438-022-01860-y

**Published:** 2022-02-25

**Authors:** Lizhu Liu, Lihong Wu, Dan Shan, Bo Han

**Affiliations:** 1 Department of Oncology, The First Affiliated Hospital of Harbin Medical University, No.23 Post Street Nangang District, Harbin, 150001 China; 2 Genecast Biotechnology Co., Ltd, Wuxi, 214104 China

**Keywords:** PDGFRA pathway, Copy number variation, Tumor-related signaling pathways, Tumor-infiltrating immune cells

## Abstract

We investigated the copy number variation (CNV) of PDGFRA pathway across all common cancer types as well as its clinical relevance. This study included a total of 10,678 patients with pan-cancerous species involving 33 types of cancers and patient information was obtained from The Cancer Genome Atlas. According to the PDGFRA pathway CNV, all samples were divided into copy number gain (CN gain) group and No CN gain group. The analysis of loss of heterozygosity (LOH) fraction, CNV burden, tumor mutation burden (TMB), and the number of immunogenic mutations were performed, as well as the correlation analysis of PDGFRA pathway CN gain with tumor-related signaling pathways and tumor-infiltrating immune cell subpopulations. The results showed that CN gain of PDGFRA pathway in the cancer patients was associated with significantly shorter overall survival. The CN gain of PDGFRA pathway was identified as a prognostic risk factor for some tumors. CN gain was accompanied by an altered percentage of LOH, CNV burden, TMB, the number of immunogenic mutations were increased and tumor-infiltrating immune cell subpopulations were less. While certain tumor-related signaling pathways, such as hypoxia, cell cycle, DNA repair, and epithelial-mesenchymal transition were more enriched in the CN gain group, quiescence, and inflammation pathways were more enriched in the No CN gain group. In conclusion, PDGFRA pathway CNV gain may be a poor prognostic factor in cancer patients.

**Supplementary Information:**

The online version contains supplementary material available at 10.1007/s00438-022-01860-y.

## Introduction

*Platelet-derived growth factor receptor alpha* (*PDGFRA*) gene encodes a tyrosine kinase receptor that activates tyrosine kinase (Ong et al. 2018). It has been shown that *PDGFRA* is involved in gene mutation, tumor cell proliferation, migration and invasion, maintenance of mesenchymal stromal cells (MSC), and immune infiltration, while it may serve as a potential biomarker and therapeutic target (Chang et al. 2018; Pantaleo et al. 2019; Wang et al. 2020a). Activation of PDGFRA signaling is sufficient to drive fibrosis in diverse organs. Targeting PDGFRA signaling can be an effective approach to treat fibrosis (Decker et al. 2017). However, the role of PDGFR signaling pathway in the pan-cancer context has yet to be investigated.

Somatic mutations are closely related to immunotherapy and clinical outcomes (e.g., survival), while these mutations can be classified as somatic point mutations (SPM) or somatic copy number variants (CNV). CNVs have been shown to be widely present in normal individuals (Hieronymus et al. 2018). As an important form of genetic structural variation, CNVs result from gains or losses of DNA segments larger than 1 kb in the human genome (Shao et al. 2019). Recently, Zheng et al. showed that CNV data had a higher validity in predicting cancer prognosis than SPM data (Zheng et al. 2020). Furthermore, the relationship between CNVs and gene expression is crucial for the prevention, diagnosis, and treatment of cancer (Shao et al. 2019). CNV-caused genetic mutations may lead to the development of immune escape (Chen et al. 2020). It has also been reported that copy number gains (CN gain) occur more frequently than copy number losses (CN loss) during epithelial-mesenchymal transition (EMT), and somatic CN gain activates gene expression by increasing gene dosage (Zhao et al. 2016). Interestingly, Anwar et al. identified frequent recurrence of CNVs in *PDGFRA* gene in a next-generation sequencing-based CNV study of invasive breast cancer patients (Anwar et al. 2020). PDGFRA CN gain was also detected in intractable epilepsy and high-grade astrocytoma and found to be significantly associated with the treatment and prognosis of the disease (Phillips et al. 2013; Vasudevaraja et al. 2021). While the importance of the tumor microenvironment has been increasingly recognized, the complexity of interaction between tumor cells and their microenvironment is becoming evident. However, characterization and clinical properties of PDGFRA CN gain in whole cancers have not been well documented.

To comprehensively characterize PDGFRA CN gain, we collected the CNV data and clinical information of pan-cancer patients from the TCGA database, systematically analyzed functional status of the tumor cells, and quantified the components of immune cells in the tumor immune microenvironment. In this study, we identified PDGFRA CN gain as a prognostic risk factor for a significantly shorter overall survival (OS) in six cancer species, including adrenocortical carcinoma (ACC), kidney renal clear cell carcinoma (KIRC), lung adenocarcinoma (LUAD), sarcoma (SARC), skin cutaneous melanoma (SKCM), and uterine corpus endometrial carcinoma (UCEC). Moreover, the enrichment of related pathways that promote tumor development was higher in these six cancer types. Loss of heterozygosity (LOH), CNV burden was higher and effector immune cells all affect patient prognosis.

## Materials and methods

### Data collection and processing

The CNV data and clinical information for 10,678 patients with pan-cancerous species were downloaded from the TCGA database (https://gdc.cancer.gov/about-data/publications/panimmune). The reference data for gene sets contained in PDGFRA signaling pathway were obtained from the GSEA website (https://www.gsea-msigdb.org/gsea/msigdb/cards/PID_PDGFRA_PATHWAY.html) (Table 1). We defined the tumor with CN gain in any of PDGFRA pathway genes as having PDGFRA pathway CN gain. Table S1 shows the number of cases for each cancer type and the number of cases with PDGFRA pathway CN gain variants in the TCGA cohort. The tumor mutation burden (TMB), the number of immunogenic mutations (TNB), CNV burden score, and LOH score were derived from the published studies (Thorsson et al. 2018).

**Table 1:** Gene list in PDGFRA pathway

Gene symbol	Ensembl Gene ID	Gene ID
FOS	ENSG00000170345	2353
IFNG	ENSG00000111537	3458
JUN	ENSG00000177606	3725
ITGAV	ENSG00000138448	3685
SRF	ENSG00000112658	6722
PDGFRA	ENSG00000134853	5156
PLCG1	ENSG00000124181	5335
ELK1	ENSG00000126767	2002
JAK1	ENSG00000162434	3716
PIK3R1	ENSG00000145675	5295
SHC1	ENSG00000160691	6464
PIK3CA	ENSG00000121879	5290
CRK	ENSG00000167193	1398
CRKL	ENSG00000099942	1399
CAV3	ENSG00000182533	859
GRB2	ENSG00000177885	2885
CSNK2A1	ENSG00000101266	1457
CAV1	ENSG00000105974	857
SOS1	ENSG00000115904	6654
RAPGEF1	ENSG00000107263	2889
SHB	ENSG00000107338	6461
SHF	ENSG00000138606	90,525

### Assessment of the functional status of tumor cells

To assess the functional status of tumor cells, 14 manually curated cancer-related signatures were calculated using the GSVA package in R software, including stemness, invasion, metastasis, proliferation, EMT, angiogenesis, apoptosis, cell cycle, differentiation, DNA damage, DNA repair, hypoxia, inflammation, and quiescence (Yuan et al. 2019).

### Quantitative analysis of tumor-infiltrating immune cell subpopulations

To quantify the components of immune cells in the tumor microenvironment, the enrichment of 28 immune cell subpopulations was assessed using the single-sample gene set enrichment analysis (ssGSEA) method (Angelova et al. 2015).

### Statistical analysis

All statistical analyses were performed using R software (version 3.4.2). Data were presented as median and interquartile variance (IQR). The Student’s *t* test and Wilcoxon rank-sum test were used to analyze the differences between the two groups for normally distributed data and non-normally distributed data, respectively. Survival data were analyzed using Kaplan–Meier (KM) survival curves and log-rank test. The relationship between PDGFRA pathway CN gain in pan-cancer species and OS was examined using univariate COX regression. Bilateral *P* values < 0.05 were considered statistically significant.

## Results

### The CNVs of PDGFRA pathway in pan-cancerous species

**Fig. 1:**
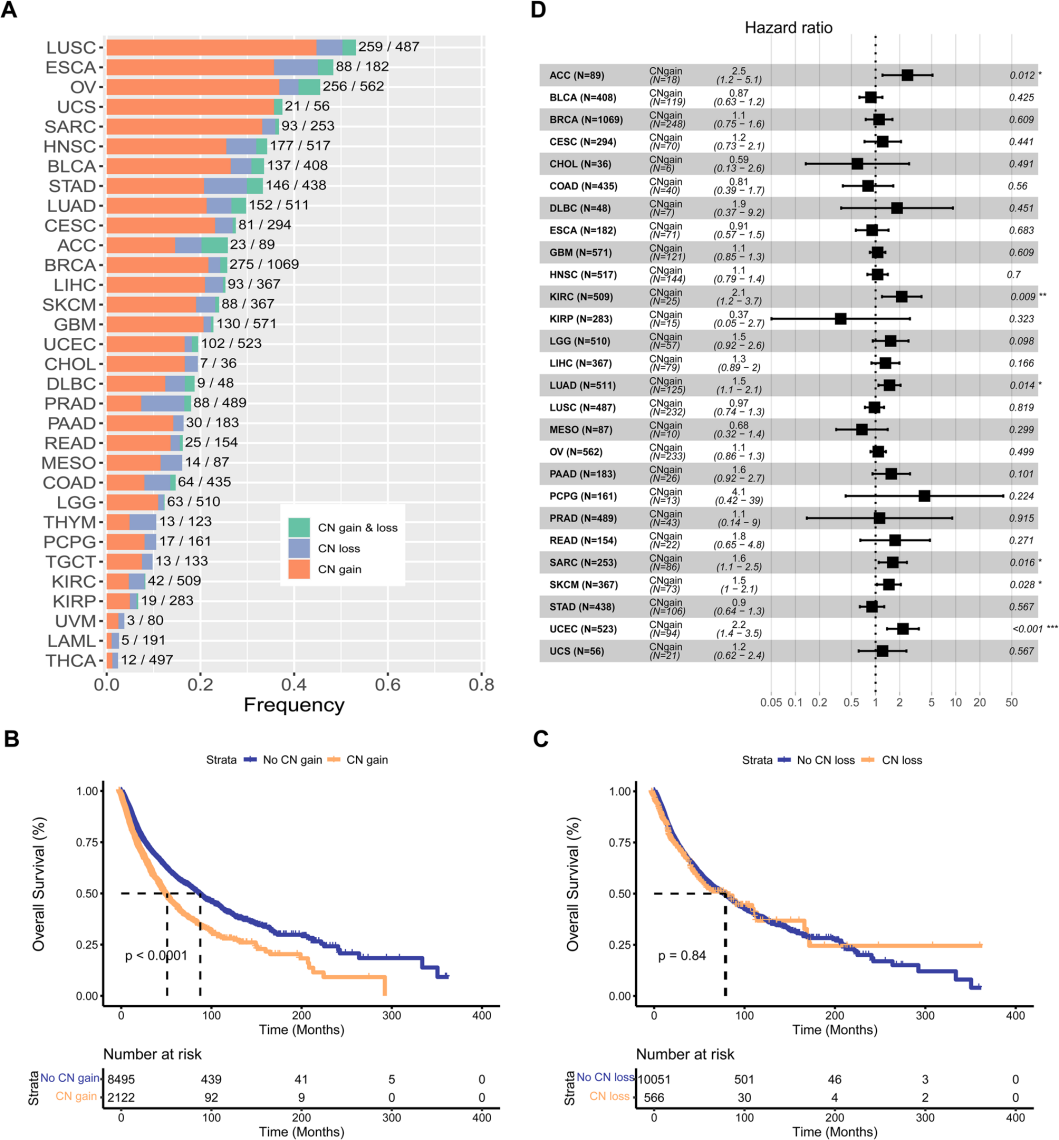
Relationships between the CN gain of PDGFRA pathway and prognosis of pan-cancer species. **A** Frequency of CNVs in PDGFRA pathway among different cancer species. The CNVs were classified as CN gain, CN loss, or CN gain & loss. **B** KM survival analysis of the relationship between the CN gain of PDGFRA pathway and OS in all pan-cancer samples. **C** KM survival analysis of the relationship between the CN loss of PDGFRA pathway and OS in all pan-cancer samples. D One-way COX regression analysis of the relationship between the CN gain and OS in various cancer species. *P* < 0.05 was considered as a significant difference. *P < 0.05, **P < 0.01, and ***P < 0.001. *ACC* adrenocortical carcinoma; *BLCA* bladder urothelial carcinoma; *BRCA* breast invasive carcinoma; *CESC* cervical squamous cell carcinoma and endocervical adenocarcinoma; *CHOL* cholangiocarcinoma; *COAD* colon adenocarcinoma; *DLBC* lymphoid neoplasm diffuse large b-cell lymphoma; *ESCA* esophageal carcinoma; *GBM* glioblastoma multiforme; *HNSC* head and neck squamous cell carcinoma; *KICH* kidney chromophobe; *KIRC* kidney renal clear cell carcinoma; *KIRP* kidney renal papillary cell carcinoma; *LAML* acute myeloid leukemia; *LGG* brain lower grade glioma; *LIHC* liver hepatocellular carcinoma; *LUAD* lung adenocarcinoma; *LUSC* lung squamous cell carcinoma; *MESO* mesothelioma; *OV* ovarian serous cystadenocarcinoma; *PAAD* pancreatic adenocarcinoma; *PCPG* pheochromocytoma and paraganglioma; *PRAD* prostate adenocarcinoma; *READ* rectum adenocarcinoma; *SARC* sarcoma; *SKCM* skin cutaneous melanoma; *STAD* stomach adenocarcinoma; *TGCT* testicular germ cell tumors; *THCA* thyroid carcinoma; *THYM* thymoma; *UCEC* uterine corpus endometrial carcinoma; *UCS* uterine carcinosarcoma; *UVM* uveal melanoma

We analyzed the CNVs of each PDGFRA pathway gene in TCGA database for each cancer type and found that the frequency of CN gain or CN loss in PDGFRA pathway varied from one cancer type to another (Fig S1). As illustrated in Fig. 1A, the frequency of CN gains in PDGFRA pathway ranged from 1 to 45% among various cancer types. Moreover, survival analysis showed that patients with CN gain in PDGFRA pathway had significantly shorter OS and worse prognosis (*P* < 0.0001) than the No CN gain group (Fig. 1B), whereas no significant correlation between CN loss in PDGFRA pathway and OS was found (Fig. 1C). Notably, univariate analysis identified PDGFRA pathway CN gain as a risk factor for poor OS in the following six cancer types: ACC (*P* < 0.05), KIRC (*P* < 0.01), LUAD (*P* < 0.05), SARC (*P* < 0.05), SKCM (*P* < 0.05), and UCEC (*P* < 0.001) (Fig. 1D).

**Fig. 2:**
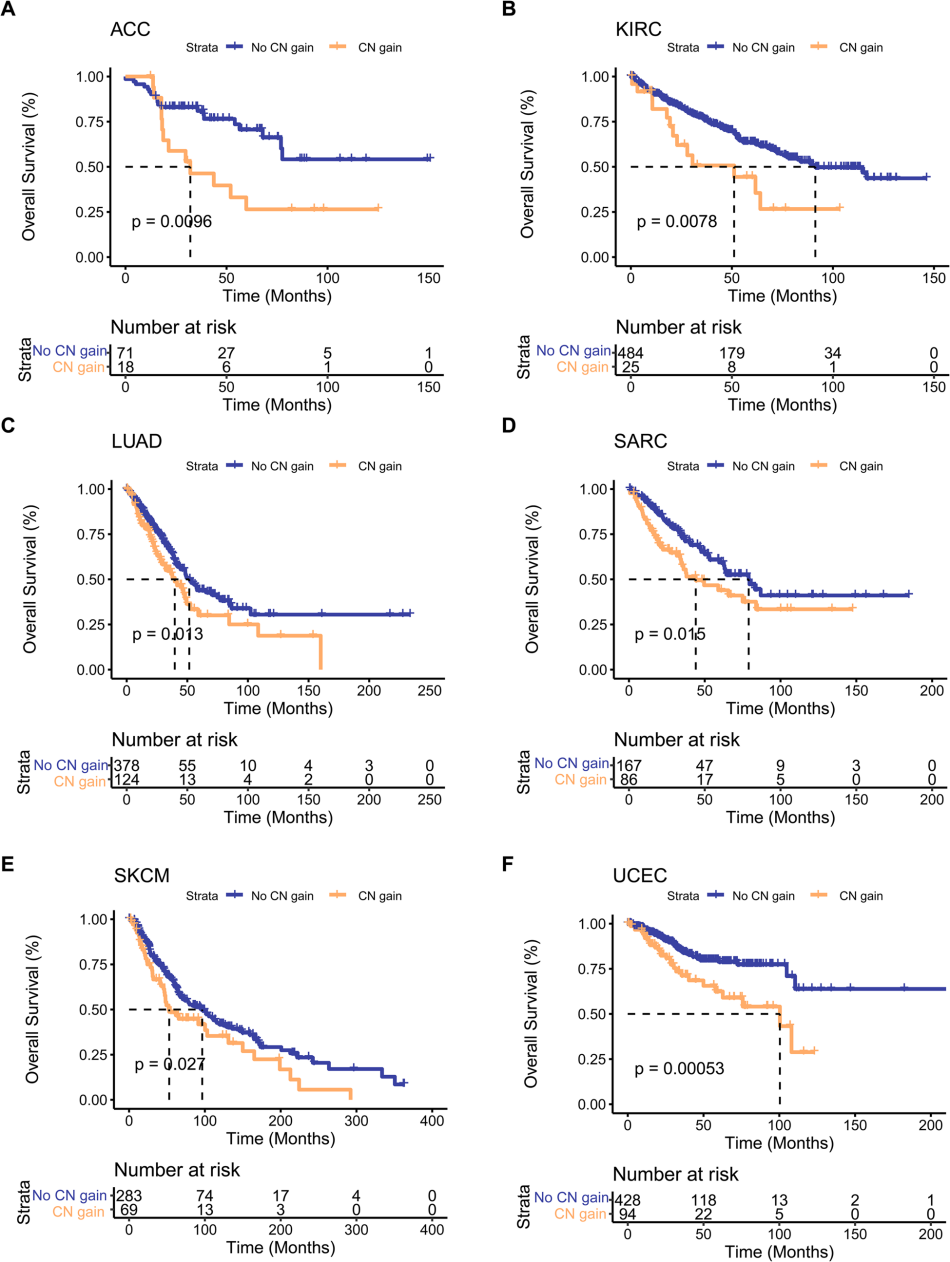
Relationship between the CN gain of PDGFRA pathway and prognosis of the six cancer types. KM survival curves showing the relationship between the CN gain of PDGFRA pathway and OS in six cancer types including ACC (**A**), KIRC (**B**), LUAD (**C**), SARC (**D**), SKCM (**E**), and UCEC (**F**)

We next performed KM survival analysis and log-rank test to verify the relationships between the CN gain and survival rates of the above-mentioned six cancer types. As shown in Fig. 2, OS was significantly shorter in patients with the CN gain of PDGFRA pathway for all six cancer types compared with the No CN gain group (*P* < 0.05).

### Analysis of PDGFRA pathway CN gain in relation to LOH fraction, CNV burden, TMB, and TNB

**Fig. 3:**
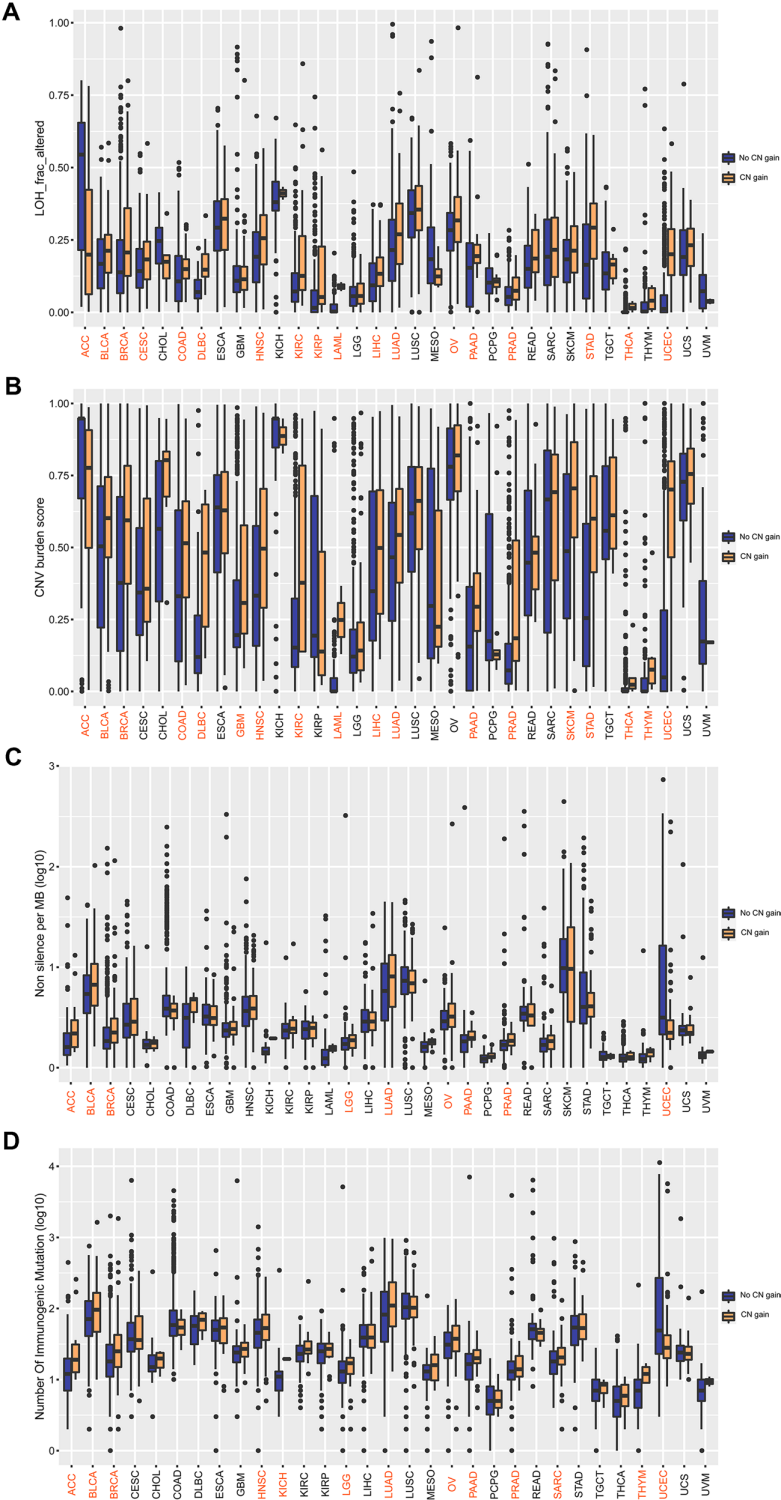
Relationships of PDGFRA pathway CN gain with LOH fraction, CNV burden, TMB, and TNB in pan-cancerous species. Comparison of LOH fraction (*A*), CNV burden (**B**), TMB (non-silence per MB) (**C**), and TNB (number of immunogenic mutation) (**D**) in different cancer species between the pathway CN gain and No CN gain groups

Based on whether a CN gain in PDGFRA pathway was identified, patients in this study were divided into two groups: CN gain and No CN gain. We further analyzed the differences in LOH fraction, CNV burden, TMB, and TNB between the two groups of patients, and found that the differences were significant (*P* < 0.05) (Fig S2 and Table S2). Moreover, we observed that the CN gain of PDGFRA pathway was associated with a higher proportion of LOH alterations and higher CNV burden in most cancers such as KIRC, LUAD, SKCM, and UCEC, while the association was in the opposite direction in ACC (Fig. 3A, B). Likewise, while association of the CN gain in PDGFRA pathway with higher TMB and TNB values was identified in ACC, LUAD and some other types of cancers, the association was in the opposite direction in UCEC (Fig. 3C,D).

### Relationships between the CN gain of PDGFRA pathway and tumor-related signaling pathways

**Fig. 4:**
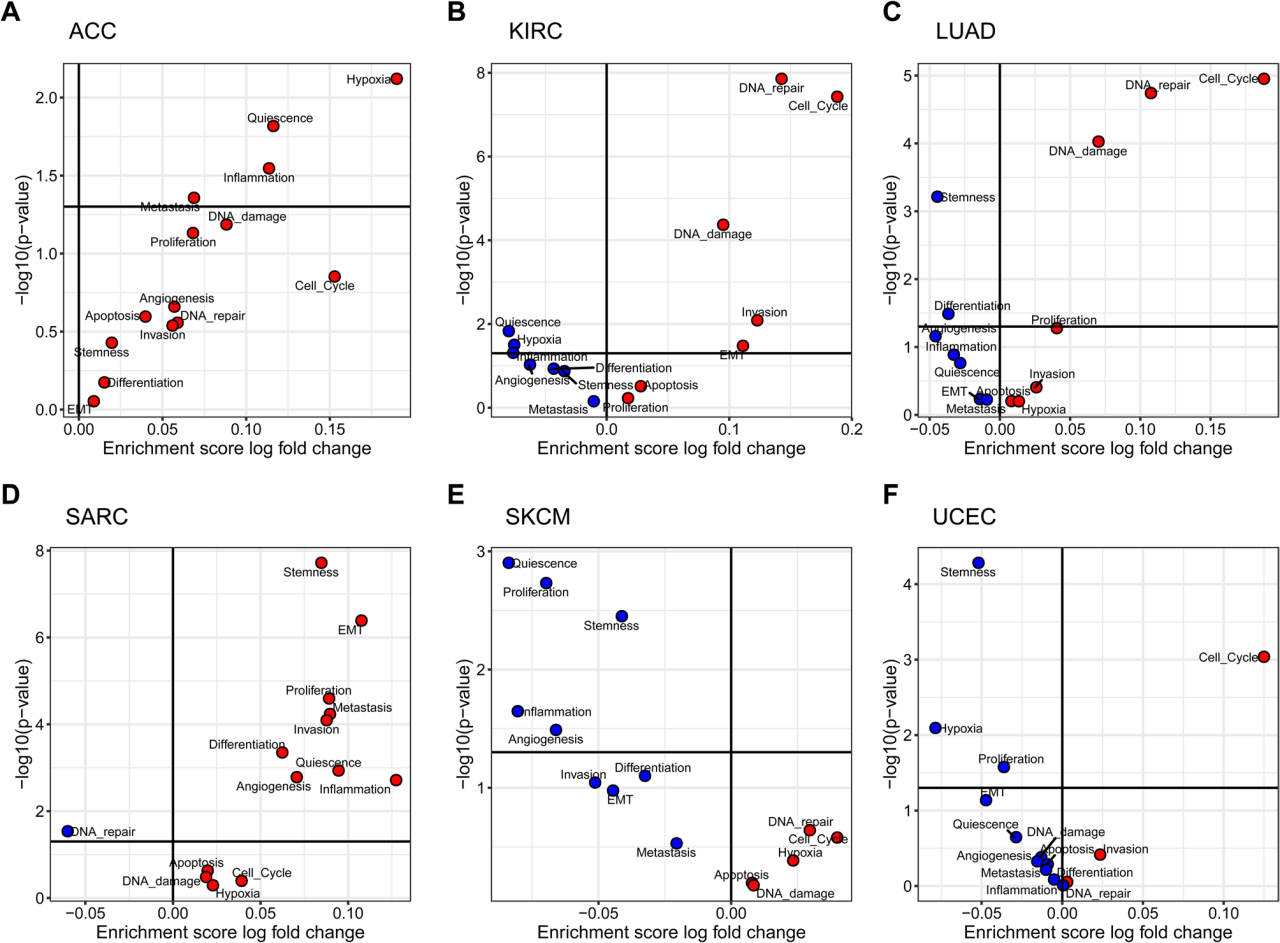
Relationships between the CN gain of PDGFRA pathway and tumor-related signaling pathways in pan-cancer species. The relationships between the CN gain of PDGFRA pathway and 14 tumor-related signaling pathways in six cancer species with significant survival, including ACC (**A**), KIRC (**B**), LUAD (**C**), SARC (**D**), SKCM (**E**), and UCEC (**F**). Note that the upper part and lower part of the horizontal divider indicate *P* < 0.05 and *P* > 0.05, respectively. The left blue dot and right red dot of the vertical divider represent CN gain/No CN gain < 1 and CN gain/No CN gain > 1, respectively

Next, we examined the relationships between the CN gain of PDGFRA pathway and 14 tumor-related signaling pathways in the pan-cancer species (Fig S3). As depicted in Fig. 4A–D, F, in ACC, KIRC, LUAD, SARC, and UCEC, pathways related to tumorigenic progression, including hypoxia, cell cycle, DNA repair, and EMT, were more enriched in the CN gain group as compared to No CN gain group. On the contrary, in SKCM, pathways such as quiescence and inflammation were more enriched in the No CN gain group (Fig. 4E).

### Relationships between the CN gain of PDGFRA pathway and tumor-infiltrating immune cell subsets

**Fig. 5:**
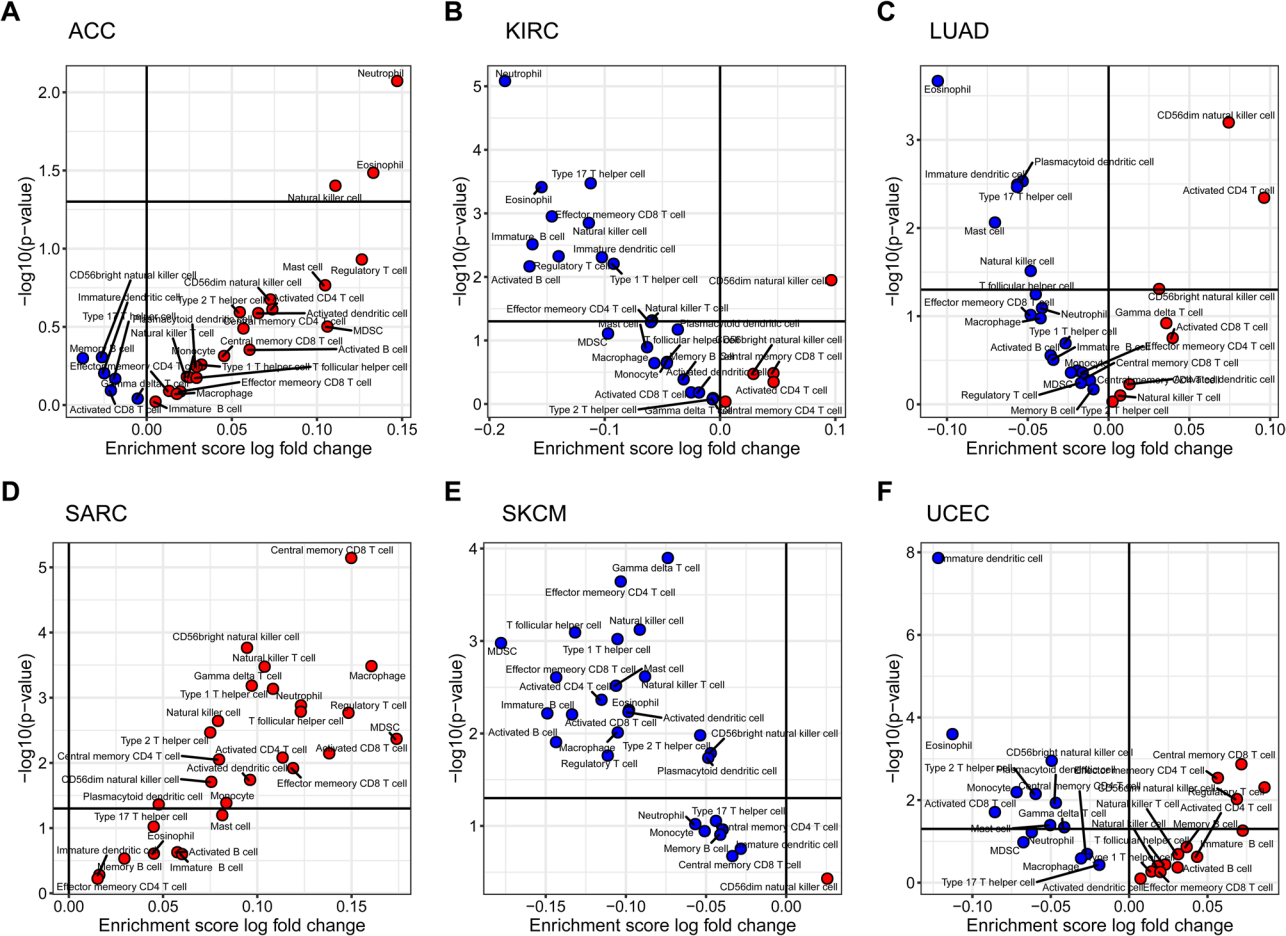
Relationships between the CN gain of PDGFRA pathway and tumor-infiltrating immune cell subpopulations in pan-cancerous species. The relationships between the CN gain of PDGFRA pathway and tumor-infiltrating immune cell subpopulations in six cancer species with significant survival, including ACC (**A**), KIRC (**B**), LUAD (**C**), SARC (**D**), SKCM (**E**), and UCEC (**F**). The upper part and lower part of the horizontal divider indicate *P* < 0.05 and *P* > 0.05, respectively. The left blue dot and right red dot of the vertical divider represent CN gain/No CN gain < 1 and CN gain/No CN gain > 1, respectively

Furthermore, we investigated the relationships between the CN gain of PDGFRA pathway and tumor-infiltrating immune cell subpopulations in pan-cancerous species (Fig. S4). Compared with the CN gain group, tumor-infiltrating immune cell subpopulations were more abundant in the No CN gain group among many cancer types. Thereafter, we analyzed the difference in the enrichment of tumor-infiltrating immune cell subpopulations between the CN gain and No CN gain groups among the six cancer species in which the CN gain of PDGFRA pathway was significantly associated with OS. The analysis revealed that in ACC, three immune cell subsets, Neutrophil, Eosinophil, and Natural killer cell, were significantly more abundant in the CN gain group (Fig. 5A), while in SARC, MDSC, Macrophage, Central memory CD8 T cell, Regulatory T cell, Activated CD8 T cell and other immune cell subsets were significantly more enriched in the CN gain group (Fig. 5D). Conversely, in KIRC and SKCM, several immune cell subsets such as Immature B cell, Activated B cell, Effector memory CD8 T cell, and Type I T helper cell were significantly more abundant in the No CN gain group (Fig. 5B, E). Notably, we observed that in LUAD and UCEC, there were significant differences in the enrichment of certain immune cell subsets such as Eosinophil between the CN gain and No CN gain groups (Fig. 5C, F).

## Discussion

*PDGFRA* is of critical importance in mesenchymal development, homeostasis and pathogenesis. Aberrant *PDGFRA* activities have been linked to a variety of diseases, including fibrosis, cancer, and pediatric diseases (Mueller et al. 2016; Decker et al. 2017). In this study, we analyzed the distribution of PDGFRA pathway CN gain in pan-cancer patients based on information from the TCGA database and investigated the association of PDGFRA pathway CN gain with tumorigenesis-related pathways, immune cell subpopulations, and survival rates.

First, we showed that the frequency of CN gain or CN loss in PDGFRA pathway varied among different cancer types. Notably, the CN gain of PDGFRA pathway was significantly associated with shorter OS in six cancer types including ACC, KIRC, LUAD, SARC, SKCM, and UCEC, thus serving as a prognostic risk factor for poor OS. It has been shown that CN gain at the *PDGFRA* locus can influence the relative frequency of tumor malignant cells and promote the tumor growth, whereas high expression of PDGFRA in glioblastoma can markedly improve the prognosis of patients (Motomura et al. 2012; Neftel et al. 2019). Combined with the previous studies, the findings in this study laterally validated the notion that signaling pathways can be selected during tumorigenesis in relation to certain cellular states during normal development and play a role in stabilizing specific malignant cellular states. LOH fraction, CNV burden, TMB, and TNB are the common types of cancer-causing mutations (Liu et al. 2019; Sokol et al. 2020). Here, we observed an increase in LOH (immune escape), CNV burden enrichment, TMB and TNB values in most of the cancer types with the CN gain of PDGFRA pathway, except in ACC and UCEC, suggesting a new research direction for ACC and UCEC.

Next, we examined the relationships between the CN gain of PDGFRA pathway and 14 tumor-related signaling pathways in each cancer type, especially the above-mentioned six cancer types with poor OS. The study revealed that there was a significant enrichment of pathways related to tumorigenic progression, such as Hypoxia, cell cycle, DNA repair, and EMT in the group of PDGFRA pathway CN gain. Hypoxia pathway is critical for the function of cells, organs and organisms. Liu et al. showed that both Hypoxia and PDGFRA amplification were associated with tumor localization and could serve as potential targets for specific therapies (Liu et al. 2016). While cell cycle plays an important regulatory role in diseases, small molecule inhibitors regulating the cell cycle can cause PDGFRA overexpression and CNV imbalance (Paugh et al. 2011). DNA repair is a key system for identifying and repairing structural or sequence abnormalities in DNA. The combination of PDGFR pathway, CNV, and DNA repair analysis can identify potential therapeutic targets (Zarghooni et al. 2010). Activation of EMT provides cancer cells with increased plasticity that is required for their invasion and metastasis. Studies have shown that PDGFRA activates the EMT pathway and decreases the expression of genes that favor epithelial integrity, enhancing metastatic diseases (Lopez-Campistrous et al. 2020). In the present study, we observed that in SKCM, quiescence and inflammation pathways were more enriched in the No CN gain group. This observation was consistent with the previous reports (Moon et al. 2017). Given that both PDGFRA CNVs and dysregulation of tumor-related pathways are usually present in cancer, we propose that the CN gain of PDGFRA pathway could be a potential target for analyzing cancer-related mechanisms in ACC, KIRC, LUAD, SARC, and UCEC.

Moreover, we found that in most cancers, tumor-infiltrating immune cell subsets, especially the effector immune cell subsets such as Activated CD8 T cell, Effector memory CD8 T cell, Activated B cell, and Immature B cell are less abundant in the group of PDGFRA pathway CN gain (Seo et al. 2018; Han et al. 2020; Bian et al. 2021). This observation may underlie the worse prognosis linked to the CN gain in PDGFRA pathway. On the contrary, in ACC and SARC, more tumor-infiltrating immune cell subsets were enriched in the CN gain group; among them, the dominant ones were pro-tumor growth cell subsets such as Neutrophil, Eosinophil, MDSC, and Macrophage. It has been shown that PDGFRA induces proliferation and differentiation of eosinophils and neutrophils, while CN gain can cause dysregulation of the two immune cell subsets (Buitenhuis et al. 2007; Wang et al. 2020b). In contrast, the effector immune cell subpopulations including Immature B cells and Activated B cells were more enriched in No CN gain group of PDGFRA pathway, particularly in KIRC and SKCM. Taken together, these data indicate that the tumor immune microenvironment varies among the cancer types and is related to CNV disorders in the PDGFRA pathway, which may require attention in subsequent immunotherapy choices.

In sum, in ACC, PDGFRA pathway CN gain promoted tumorigenesis, leading to a poor survival, which was found to be significantly associated with ACC in our study for the first time. The CN gain of PDGFRA pathway in KIRC, LUAD and UCEC leaded to a higher proportion of LOH occurrence and enrichment of CNV burden, with less abundant effector immune cells, promoting tumorigenesis related to worse survival. Previous studies have shown that PDGFRA is associated with KIRC protein expression, gene mutations and brain metastasis, while affecting the survival of patients (Terada 2012; Schiefer et al. 2015). PDGFRA mutations have also been detected in LUAD (Seo et al. 2012). In addition, Wang et al. reported that CNV has a significant negative impact on the survival rate of UCEC patients (Wang et al. 2020c). However, the specific mechanisms underlying the CN gain of PDGFRA pathway and the above three cancer types remain to be further investigated. In SARC, the CN gain of PDGFRA pathway promoted tumorigenesis. The tumor-related pathways and immune cell subpopulations were relatively enriched, but the immune cells had weaker effects, resulting in worse survival rate. CNV is an important event in the development of SARC and is associated with dysregulation of PDGFRA, indicating a poor prognosis (Helbig et al. 2017). This observation was consistent with the OS data obtained in the present study. Similarly, we showed that in SKCM, the CN gain of PDGFRA pathway caused a lower enrichment of multiple immune cell subpopulations, resulting in worse survival. It has been demonstrated that while the immune microenvironment is critical to the treatment of SKCM patients, CNV has the potential of being a biomarker for active melanoma disease and survival (Silva et al. 2018; Pozniak et al. 2019). Overall, the present study not only prove the previous observations, but also provide new insights into mechanisms underlying the effects of PDGFRA pathway CN gain on the immune microenvironment.

In conclusion, the CN gain of PDGFRA pathway was identified as a prognostic risk factor for six cancer types: ACC, KIRC, LUAD, SARC, SKCM, and UCEC. The presence of PDGFRA pathway CN gain in the tumors was associated with significantly shorter OS of patients. This study suggested that the impact of tumor characteristics and immune characteristics on the survival of patients may vary from one tumor type to another. The presence of PDGFRA pathway CN gain in tumors with enriched pathways favoring tumorigenic progression. Patients with LOH for immune escape, high enrichment of CNV burden, and few abundant effector immune cells have poorer prognosis. The underlying mechanisms remain to be further investigated.

## Supplementary Information

Below is the link to the electronic supplementary material.

Supplementary file1 Supplementary Figure 1. CNVs of each PDGFRA pathway gene in pan-cancerous species. The copy number variation in different cancer species was plotted by the proportion of the CN gain and CN loss of each gene in the population. The darkest color indicates the proportion of 10% in the population. (TIF 9309 KB)

Supplementary file2 Supplementary Figure 2. Relationships of the CN gain of PDGFRA pathway with LOH fraction, CNV burden, TMB, and TNB in all pan-cancer samples. Comparison of LOH fraction (A), CNV burden (B), TMB (nonsilence per MB) (C), and TNB (number of immunogenic mutation) (D) in all pan-cancer samples between the CN gain group and No CN gain group. (TIF 7065 KB)

Supplementary file3 Supplementary Figure 3. Differences in the enrichment of tumor-related signaling pathways between the CN gain and No CN gain groups among various cancer types. Red bubbles represent a higher enrichment of the signaling pathway in the CN gain group compared with No CN gain group, while blue bubbles indicate a lower enrichment of the pathway in the CN gain group as compared to No CN gain group. The bubble size and black bubble edges represent |log2Foldchange| and P < 0.05, respectively. (TIF 4514 KB)

Supplementary file4 Supplementary Figure 4. Differences in the enrichment of immune cell subsets in different cancer species between the CN gain and No CN gain groups. The red bubbles represent a higher enrichment of the immune cell subpopulation in the CN gain group compared with No CN gain group, while the blue bubbles indicate a lower enrichment of the subset in the CN gain group as compared to No CN gain group. The bubble size and black bubble edges represent |log2Foldchange| and P < 0.05, respectively. (TIF 8008 KB)

Supplementary file5 (DOCX 21 KB)

## Data Availability

The datasets used during the current study available from the corresponding author on reasonable request.

## Abbreviations

PDGFRA: Platelet-derived growth factor receptor alpha
MSC: Mesenchymal stromal cells
SPM: Somatic point mutations
CNV: Copy number variants
EMT: Epithelial-mesenchymal transition
OS: Overall survival
ACC: Adrenocortical carcinoma
KIRC: Kidney renal clear cell carcinoma
LUAD: Lung adenocarcinoma
SARC: Sarcoma
SKCM: Skin Cutaneous Melanoma
UCEC: Uterine Corpus Endometrial Carcinoma
LOH: Loss of heterozygosity
TMB: Tumor mutation burden
TNB: Tumor neoantigen burden
ssGSEA: single-sample gene set enrichment analysis
IQR: Interquartile variance
KM: Kaplan–Meier
