# Supplementary figures and images for: Characterization and clinical relevance of PDGFRA pathway copy number variation gains across human cancers
Source: Mol Genet Genomics. 2022 Feb 25;297(2):561–71. doi: 10.1007/s00438-022-01860-y (PMC8960564; doi:10.1007/s00438-022-01860-y)

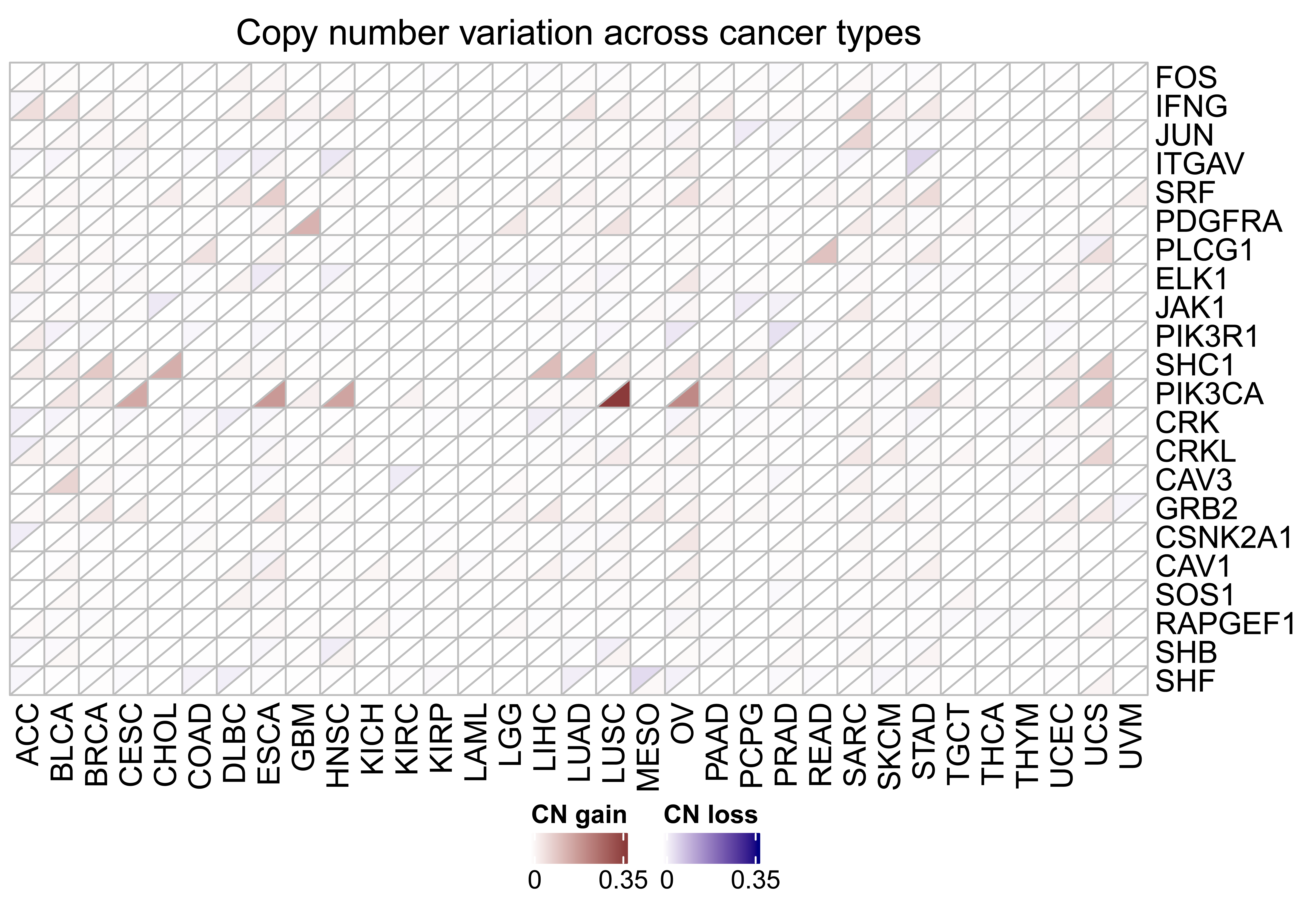

Supplement: Supplementary file 1 — Supplementary file1 Supplementary Figure 1. CNVs of each PDGFRA pathway gene in pan-cancerous species. The copy number variation in different cancer species was plotted by the proportion of the CN gain and CN loss of each gene in the population. The darkest color indicates the proportion of 10% in the population. (TIF 9309 KB) [file 438_2022_1860_MOESM1_ESM.tif]

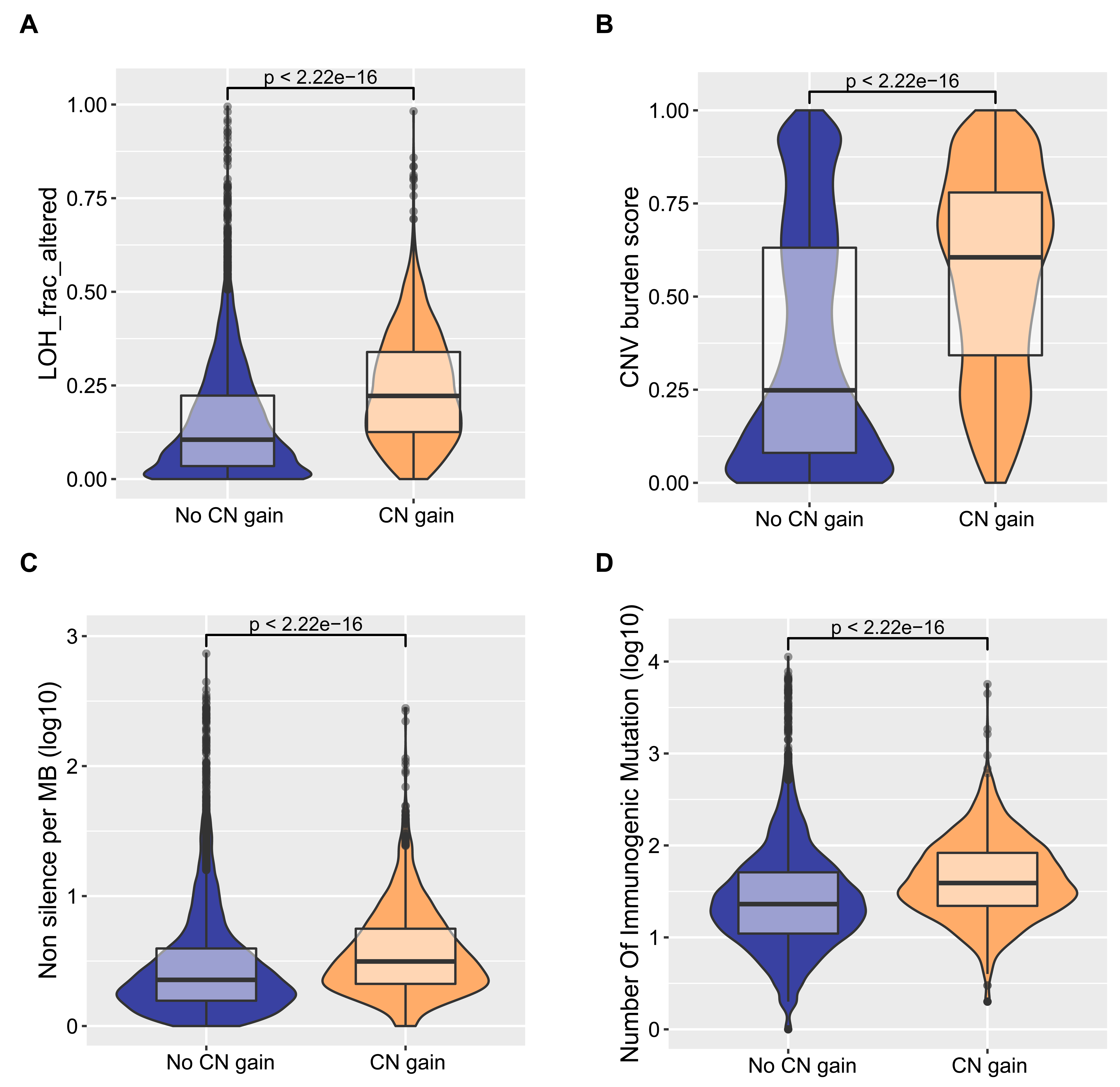

Supplement: Supplementary file 2 — Supplementary file2 Supplementary Figure 2. Relationships of the CN gain of PDGFRA pathway with LOH fraction, CNV burden, TMB, and TNB in all pan-cancer samples. Comparison of LOH fraction (A), CNV burden (B), TMB (nonsilence per MB) (C), and TNB (number of immunogenic mutation) (D) in all pan-cancer samples between the CN gain group and No CN gain group. (TIF 7065 KB) [file 438_2022_1860_MOESM2_ESM.tif]

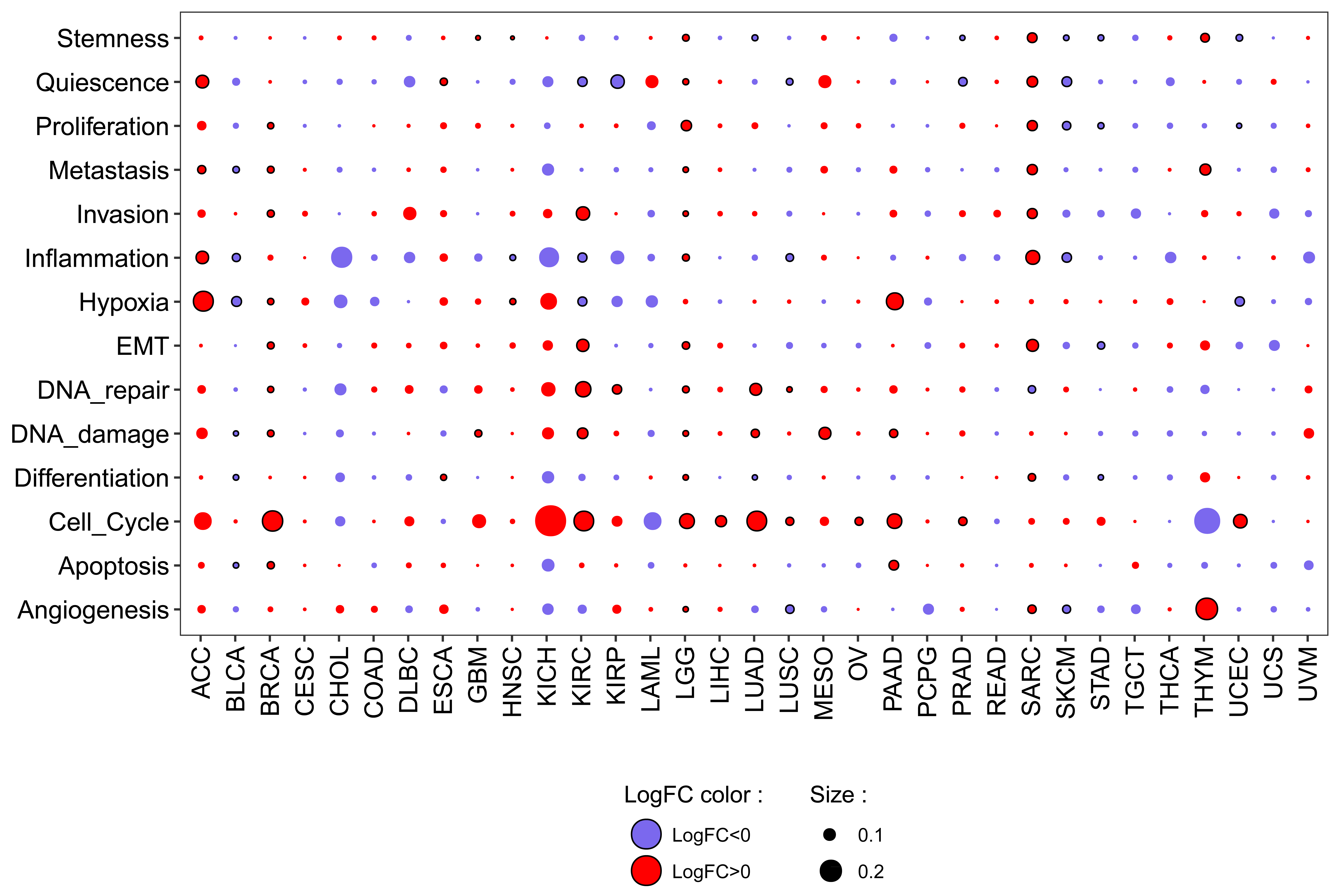

Supplement: Supplementary file 3 — Supplementary file3 Supplementary Figure 3. Differences in the enrichment of tumor-related signaling pathways between the CN gain and No CN gain groups among various cancer types. Red bubbles represent a higher enrichment of the signaling pathway in the CN gain group compared with No CN gain group, while blue bubbles indicate a lower enrichment of the pathway in the CN gain group as compared to No CN gain group. The bubble size and black bubble edges represent |log2Foldchange| and P < 0.05, respectively. (TIF 4514 KB) [file 438_2022_1860_MOESM3_ESM.tif]

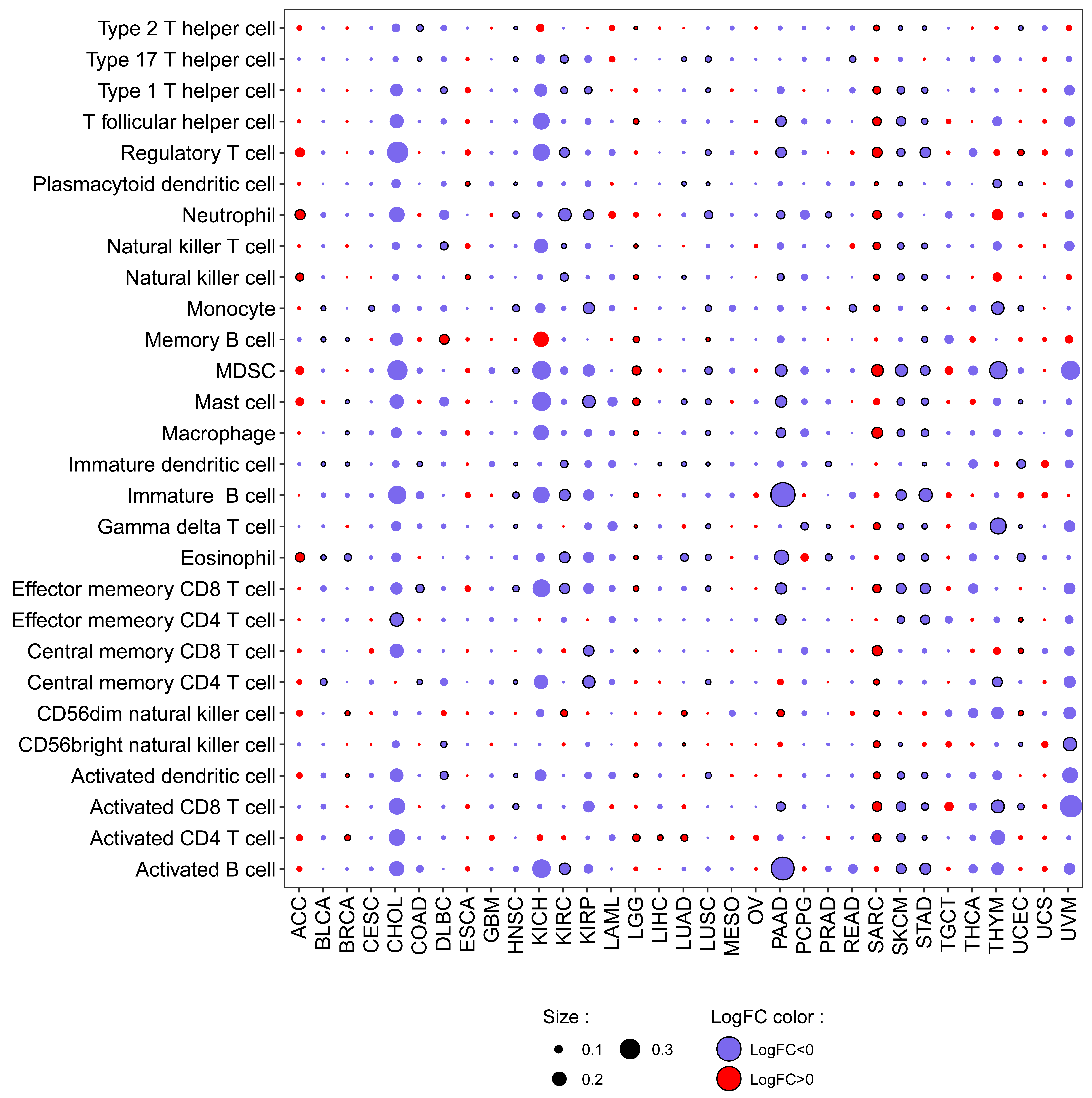

Supplement: Supplementary file 4 — Supplementary file4 Supplementary Figure 4. Differences in the enrichment of immune cell subsets in different cancer species between the CN gain and No CN gain groups. The red bubbles represent a higher enrichment of the immune cell subpopulation in the CN gain group compared with No CN gain group, while the blue bubbles indicate a lower enrichment of the subset in the CN gain group as compared to No CN gain group. The bubble size and black bubble edges represent |log2Foldchange| and P < 0.05, respectively. (TIF 8008 KB) [file 438_2022_1860_MOESM4_ESM.tif]
