# Supplementary material for: Characterization and clinical relevance of PDGFRA pathway copy number variation gains across human cancers
Source: Mol Genet Genomics. 2022 Feb 25;297(2):561–71. doi: 10.1007/s00438-022-01860-y (PMC8960564; doi:10.1007/s00438-022-01860-y)
Supplement: Supplementary file 5 — Supplementary file5 (DOCX 21 KB) [file 438_2022_1860_MOESM5_ESM.docx]

**Table S1 Numbers of patients analyzed in this study**

| **Cancer type** | **No. of total patients** | **No. of patients with CN gain** |
| --- | --- | --- |
| ACC | 89 | 18 |
| BLCA | 408 | 119 |
| BRCA | 1069 | 248 |
| CESC | 294 | 70 |
| CHOL | 36 | 6 |
| COAD | 435 | 40 |
| DLBC | 48 | 7 |
| ESCA | 182 | 71 |
| GBM | 571 | 121 |
| HNSC | 517 | 144 |
| KICH | 65 | 2 |
| KIRC | 509 | 25 |
| KIRP | 283 | 15 |
| LAML | 191 | 2 |
| LGG | 510 | 57 |
| LIHC | 367 | 79 |
| LUAD | 511 | 125 |
| LUSC | 487 | 232 |
| MESO | 87 | 10 |
| OV | 562 | 233 |
| PAAD | 183 | 26 |
| PCPG | 161 | 13 |
| PRAD | 489 | 43 |
| READ | 154 | 22 |
| SARC | 253 | 86 |
| SKCM | 367 | 73 |
| STAD | 438 | 106 |
| TGCT | 133 | 10 |
| THCA | 497 | 6 |
| THYM | 123 | 6 |
| UCEC | 523 | 94 |
| UCS | 56 | 21 |
| UVM | 80 | 2 |

ACC: Adrenocortical carcinoma; BLCA: Bladder Urothelial Carcinoma; BRCA: Breast invasive carcinoma; CESC: Cervical squamous cell carcinoma and endocervical adenocarcinoma; CHOL: Cholangiocarcinoma; COAD: Colon adenocarcinoma; DLBC: Lymphoid Neoplasm Diffuse Large B-cell Lymphoma; ESCA: Esophageal carcinoma; GBM: Glioblastoma multiforme; HNSC: Head and Neck squamous cell carcinoma; KICH: Kidney Chromophobe; KIRC: Kidney renal clear cell carcinoma; KIRP: Kidney renal papillary cell carcinoma; LAML: Acute Myeloid Leukemia; LGG: Brain Lower Grade Glioma; LIHC: Liver hepatocellular carcinoma; LUAD: Lung adenocarcinoma; LUSC: Lung squamous cell carcinoma; MESO: Mesothelioma; OV: Ovarian serous cystadenocarcinoma; PAAD: Pancreatic adenocarcinoma; PCPG: Pheochromocytoma and Paraganglioma; PRAD: Prostate adenocarcinoma; READ: Rectum adenocarcinoma; SARC: Sarcoma; SKCM: Skin Cutaneous Melanoma; STAD: Stomach adenocarcinoma; TGCT: Testicular Germ Cell Tumors; THCA: Thyroid carcinoma; THYM: Thymoma; UCEC: Uterine Corpus Endometrial Carcinoma; UCS: Uterine Carcinosarcoma; UVM: Uveal Melanoma

**Table S2 Statistical results of LOH, CNV burden, TMB and TNB between CN gain and No CN gain in PDGFRA pathway in each cancer types, respectively**

| **Cancer type** | **LOH P value** | **CNV burden P value** | **TMB P value** | **TNB P value** |
| --- | --- | --- | --- | --- |
| ACC | 0.00354 | 0.0162 | 0.0166 | 0.0237 |
| BLCA | 0.00467 | 0.000418 | 0.00768 | 0.00896 |
| BRCA | 6.86E-12 | 1.02E-12 | 6.10E-09 | 5.20E-07 |
| CESC | 0.0067 | 0.174 | 0.673 | 0.422 |
| CHOL | 0.217 | 0.217 | 0.984 | 0.482 |
| COAD | 0.009 | 0.00248 | 0.0693 | 0.237 |
| DLBC | 0.00437 | 0.0155 | 0.15 | 0.471 |
| ESCA | 0.694 | 0.496 | 0.656 | 0.596 |
| GBM | 0.34 | 3.10E-06 | 0.411 | 0.0592 |
| HNSC | 4.72E-07 | 1.09E-07 | 0.136 | 0.0308 |
| KICH | 0.662 | 0.718 | 0.0551 | 0.0416 |
| KIRC | 0.00159 | 0.00187 | 0.159 | 0.0777 |
| KIRP | 0.0198 | 0.0634 | 0.983 | 0.447 |
| LAML | 0.0382 | 0.0313 | 0.225 | - |
| LGG | 0.901 | 0.266 | 0.031 | 0.00976 |
| LIHC | 0.000131 | 0.0114 | 0.995 | 0.772 |
| LUAD | 0.000825 | 0.00469 | 0.00529 | 0.0022 |
| LUSC | 0.0877 | 0.0976 | 0.707 | 0.686 |
| MESO | 0.143 | 0.91 | 0.0882 | 0.32 |
| OV | 0.000738 | 0.0708 | 0.0067 | 0.0114 |
| PAAD | 0.0329 | 0.00333 | 0.00484 | 0.0394 |
| PCPG | 0.92 | 0.223 | 0.0829 | 0.513 |
| PRAD | 0.00632 | 1.36E-07 | 0.000397 | 0.0358 |
| READ | 0.098 | 0.79 | 0.42 | 0.261 |
| SARC | 0.808 | 0.608 | 0.0802 | 0.0425 |
| SKCM | 0.0516 | 4.56E-05 | 0.388 | - |
| STAD | 1.94E-08 | 9.88E-13 | 0.886 | 0.69 |
| TGCT | 0.341 | 0.508 | 0.99 | 0.621 |
| THCA | 0.0307 | 0.0164 | 0.392 | 0.732 |
| THYM | 0.0837 | 0.0321 | 0.0753 | 0.0215 |
| UCEC | 6.89E-26 | 7.19E-23 | 5.69E-08 | 3.37E-06 |
| UCS | 0.338 | 0.603 | 0.854 | 0.621 |
| UVM | 0.528 | 0.963 | 0.19 | 0.275 |

ACC: Adrenocortical carcinoma; BLCA: Bladder Urothelial Carcinoma; BRCA: Breast invasive carcinoma; CESC: Cervical squamous cell carcinoma and endocervical adenocarcinoma; CHOL: Cholangiocarcinoma; COAD: Colon adenocarcinoma; DLBC: Lymphoid Neoplasm Diffuse Large B-cell Lymphoma; ESCA: Esophageal carcinoma; GBM: Glioblastoma multiforme; HNSC: Head and Neck squamous cell carcinoma; KICH: Kidney Chromophobe; KIRC: Kidney renal clear cell carcinoma; KIRP: Kidney renal papillary cell carcinoma; LAML: Acute Myeloid Leukemia; LGG: Brain Lower Grade Glioma; LIHC: Liver hepatocellular carcinoma; LUAD: Lung adenocarcinoma; LUSC: Lung squamous cell carcinoma; MESO: Mesothelioma; OV: Ovarian serous cystadenocarcinoma; PAAD: Pancreatic adenocarcinoma; PCPG: Pheochromocytoma and Paraganglioma; PRAD: Prostate adenocarcinoma; READ: Rectum adenocarcinoma; SARC: Sarcoma; SKCM: Skin Cutaneous Melanoma; STAD: Stomach adenocarcinoma; TGCT: Testicular Germ Cell Tumors; THCA: Thyroid carcinoma; THYM: Thymoma; UCEC: Uterine Corpus Endometrial Carcinoma; UCS: Uterine Carcinosarcoma; UVM: Uveal Melanoma
